# The biochemically defined super relaxed state of myosin—A paradox

**DOI:** 10.1016/j.jbc.2023.105565

**Published:** 2023-12-14

**Authors:** Saffie Mohran, Kristina Kooiker, Max Mahoney-Schaefer, Christian Mandrycky, Kerry Kao, An-Yue Tu, Jeremy Freeman, Farid Moussavi-Harami, Michael Geeves, Michael Regnier

**Affiliations:** 1 Department of Bioengineering, University of Washington, Seattle, Washington, USA; 2 Center for Translational Muscle Research, University of Washington, Seattle, Washington, USA; 3 Division of Cardiology, University of Washington, Seattle, Washington, USA; 4 School of Biosciences, University of Kent, Canterbury, UK

**Keywords:** myosin regulation, thick filament regulation, ATPase, cardiomyopathy, mavacamten, deoxyATP, super-relaxed state

## Abstract

The biochemical SRX (super-relaxed) state of myosin has been defined as a low ATPase activity state. This state can conserve energy when the myosin is not recruited for muscle contraction. The SRX state has been correlated with a structurally defined ordered (*versus* disordered) state of muscle thick filaments. The two states may be linked *via* a common interacting head motif (IHM) where the two heads of heavy meromyosin (HMM), or myosin, fold back onto each other and form additional contacts with S2 and the thick filament. Experimental observations of the SRX, IHM, and the ordered form of thick filaments, however, do not always agree, and result in a series of unresolved paradoxes. To address these paradoxes, we have reexamined the biochemical measurements of the SRX state for porcine cardiac HMM. In our hands, the commonly employed mantATP displacement assay was unable to quantify the population of the SRX state with all data fitting very well by a single exponential. We further show that mavacamten inhibits the basal ATPases of both porcine ventricle HMM and S1 (K_i_, 0.32 and 1.76 μM respectively) while dATP activates HMM cooperatively without any evidence of an SRX state. A combination of our experimental observations and theories suggests that the displacement of mantATP in purified proteins is not a reliable assay to quantify the SRX population. This means that while the structurally defined IHM and ordered thick filaments clearly exist, great care must be employed when using the mantATP displacement assay.

Current views suggest that the interaction between actin and myosin in striated muscle is regulated by both thin and thick filament mechanisms. Thin filament activation is well-studied and describes how the presence of calcium modulates the access of myosin binding sites on actin through troponin and tropomyosin interactions (1). Regulation of myosin, and the availability of actively cycling heads within the thick filament system, remains a subject of intense study (2, 3, 4, 5, 6, 7). Evidence for myosin regulation comes from structural studies utilizing x-ray diffraction or high-resolution cryo-EM, or biochemical studies using nucleotide displacement assays.

X-ray diffraction studies have identified changes in the equatorial and meridional reflections of striated muscle that can describe an order-disorder transition of myosin heads along the thick filament (8) accompanying muscle activation. These observations agree with cryo-EM measurements that describe populations of myosin heads that are either available for binding to thin filaments and crossbridge cycling or in a structurally sequestered, folded-back state. This folded-back state includes the interacting head motif (IHM), which describes when the two heads of a myosin in the M.ADP.Pi state combine and fold back onto S2 to inhibit each other. The IHM has been identified in almost all 2-headed myosin types by assessing both isolated myosin and myosin within a thick filament (9). The IHM conformation has been previously well studied in smooth muscle and molluscan striated muscle myosin (10, 11, 12, 13). Recent high resolution cryo-EM studies have resolved the detailed structure of the IHM for an isolated human cardiac heavy meromyosin (HMM) (14) and in the C-zone of relaxed muscle fibres (15, 16).

A transition between two myosin states has also been described biochemically by observing the rate of ATP turnover by myosin heads in a variety of preparations including HMM, full-length myosin, synthetic thick filaments, myofibrils, and cells (17, 18, 19). These nucleotide displacement assays have led to the biochemical definition of a super-relaxed state (SRX) (3, 17, 18, 20, 21, 22, 23) with an ATP turnover rate less than 1/10th of the standard, disordered relaxed (DRX) state of myosin (∼0.02 s^−1^), (2, 23, 24). Previous studies have observed that the SRX and DRX states of myosin can be manipulated by temperature, changes in ionic strength, phosphorylation of both regulatory light chains (RLCs) and myosin binding protein-C (MyBP-C), and treatment with various small molecules (*e.g.*, mavacamten, blebbistatin, and dATP) (17, 25). This is consistent with the two states being in thermodynamic equilibrium which is readily perturbed by these conditions and/or treatments.

A major debate within the field questions if the biochemically defined SRX state and structural definitions of ordered myosin and IHM are all manifestations of the same regulatory mechanism of striated muscle myosin. In a recent review, Craig and Padron, 2022, concluded that they were similar, however, several recent publications have described discrepancies between populations of myosin in the biochemically defined SRX state and the structurally defined ordered or IHM conformation (17, 26, 27). These reports have led to speculation about different subtypes of myosin head conformations and potential differences in the IHM position where one myosin head remains sequestered along the S2/thick filament backbone while the second is liberated into the pool of more rapidly cycling myosin heads. These unknowns leave the field in a state of some confusion, as different experimental assays measure distinct aspects of myosin structure and function. It is important to understand what each assay observes, and how *in-vitro* studies of purified myosin proteins are comparable to intact myofibril and myocyte preparations.

The standard biochemical SRX assay (nucleotide displacement) mixes the fluorescent ATP analogue, mantATP, with myosin preparations ranging from purified myosin and its subfragments (S1, HMM) to myofibrils and whole myocytes (2, 23). Once steady state binding is established, the addition of ∼100-fold excess unlabeled ATP is utilized to chase off the mantATP. Previous reports have described the observed fluorescent decay as a double exponential. The rate constant of the initial, fast phase of the double exponential fluorescence decay (∼0.02–0.05 s^−1^) is consistent with the ATPase rate of isolated myosin heads. The second, slower phase (∼0.002–0.005 s^−1^) is defined as the SRX population. This SRX population has been assumed by many to be the IHM state of myosin.

As set out below, there is a set of paradoxes associated with utilizing the mantATP displacement assay to quantify the SRX state. The SRX/DRX transition is usually assumed to be at thermodynamic equilibrium, where the equilibrium position of the SRX/DRX population can be readily manipulated through conditions listed in Figure 1. This equilibrium definition, however, contradicts the reported experimental data. A model with the SRX and DRX at equilibrium would be expected to generate a single exponential displacement reaction for mantATP, as seen in scallop myosin which is regulated by calcium binding to the essential light chain (10, 28). A single exponential displacement is expected because the nucleotide bound in the SRX state can escape *via* the turnover of the SRX state ([SRX] *k*_SRX_) or *via* a transition to the DRX state. A double exponential can only be generated if the interconversion of the DRX and SRX state is slower than the turnover within the SRX state (*k*_SRX_). This would mean that the two populations are kinetically isolated on the time scale of the slow phase (>100 s). Such a condition has been described in smooth muscle myosin, where a mixture of phosphorylated and dephosphorylated populations of myosin do not interconvert in the absence of a kinase/phosphatase.

**Figure 1:**
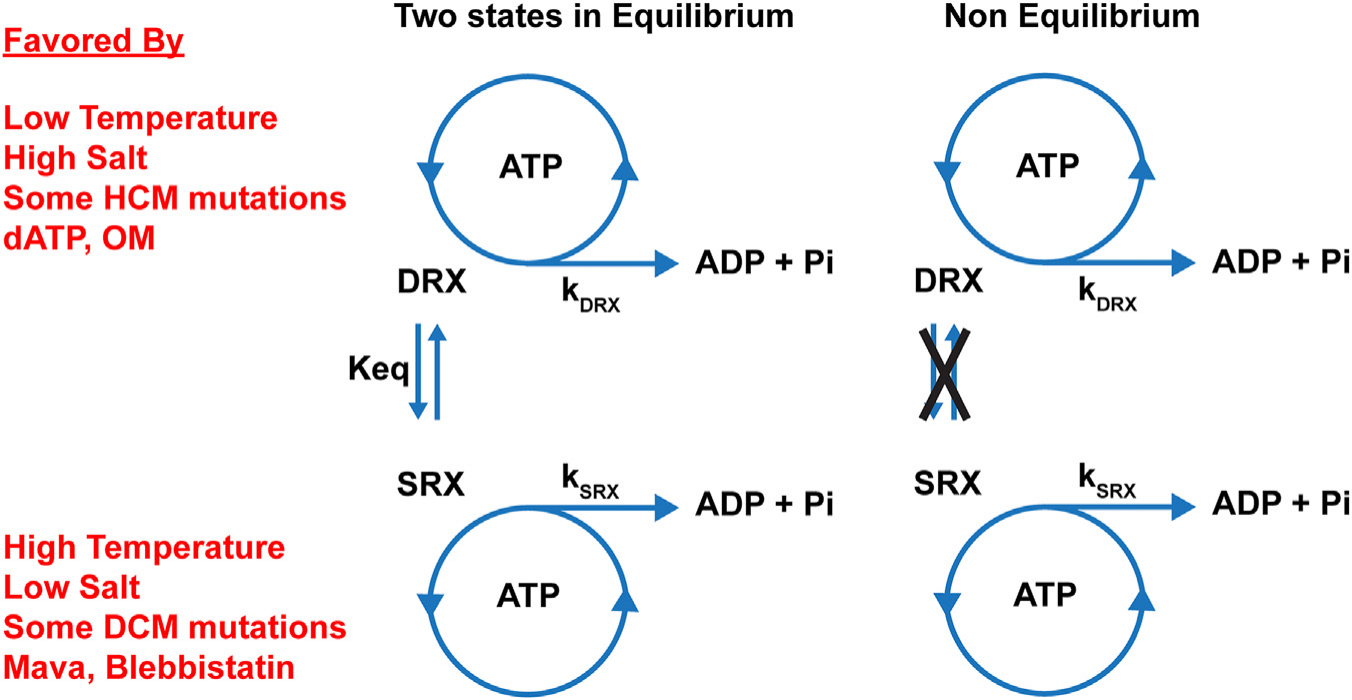
**Equilibrium and nonequilibrium models of the SRX and DRX states of myosin**. In the two states in the equilibrium model, the DRX and SRX can interconvert to reach equilibrium on the second time scale. The items listed in *red* can bias the equilibrium position toward the state they are next to. In the nonequilibrium model, the two states do not interconvert on a time scale less than several hundred seconds.

A second paradox of the mantATP nucleotide displacement assay and the quantification of the SRX state in isolated myosin preparations is that rarely do steady-state ATPase assays agree with the predictions of the SRX and DRX properties. Mavacamten (Mava) has been widely used as an inhibitor of myosin to increase the structurally ordered state of myosin by organizing the myosin heads along the thick filament backbone (20). In addition to the structural definition, it has also been proposed that Mava regulates myosin function by increasing the biochemical SRX population (3). Gollapudi *et al.* (18), showed that the basal ATPase rates measured for bovine cardiac S1, HMM, and synthetic thick filaments (∼0.015–0.017 s^−1^) were all inhibited by Mava to ∼30% of the basal ATPase rate (K_i_ ∼0.6–0.8 μM). However, if Mava stabilizes the biochemical SRX state, it might be expected to reduce ATPase about 10-fold, and to interact quite differently with single headed S1 compared with two-headed HMM or myosin in a thick filament where additional head-head and head-backbone stabilizing interactions are possible.

In a follow up study, Gollapudi *et al.* (29), reported that Mava, in addition to inhibiting the ATPase of bovine synthetic thick filament, also increased the SRX population from 10% of the myosin in basal conditions to 100% in saturating Mava concentrations. The data predict >90% inhibition of the steady state ATPase. This is not compatible with the much smaller (to 30%) inhibition of the ATPase activity reported by the same authors.

A third paradox addresses how rapidly the SRX state is formed. In myofibril measurements performed by our group, Walklate *et al.* (17) describes the SRX state forming within 200 ms of mantATP binding to myosin in rigor conditions. The SRX is therefore formed very quickly, is stable for hundreds of seconds, and yet, only accounts for ∼30% of the myosin head population. This percentage of the myosin population pool is not compatible with an equilibrium mixture of SRX/DRX where K_eq_ = rate of decay of SRX/rate of formation of SRX.

While these paradoxes remain unresolved, the interpretation of the SRX state in isolated protein assays remains in doubt. To address this, we returned to the original mantATP chase assay using HMM prepared from porcine ventricle muscle. To our surprise, we found no evidence of a secondary slow phase in our purified protein assays. The introduction of Mava and other myosin modulators also did not introduce a second phase, with all reactions expressing a single exponential mantATP fluorescent decay. With only a single exponential, the assay provides no estimate of the SRX to DRX ratio within the HMM population as it is currently defined. It is important to state here that we do not dispute the structural evidence that myosin alone and in thick filaments can exist in two forms (as originally defined for smooth muscle HMM and scallop HMM), but that the current mantATP displacement assays utilized to quantify the SRX population requires careful reevaluation.

## Results

The standard mantATP displacement assay was performed using porcine cardiac HMM (pc-HMM). A total of 125 nM pc-HMM was mixed with 2 μM mantATP and allowed to react for 1 min to achieve steady state before rapidly mixing with an excess of unlabeled ATP (125 μM) in a stopped flow fluorimeter. Note, all concentrations refer to the concentrations after final mixing in the stopped flow observation chamber. Great caution was taken to ensure stable baselines over the 3 to 5 min typically used to record the data as set out in the Experimental procedures section.

### MantATP displacement assay using pc-HMM resulted in a single exponential fluorescent decay

**Figure 2:**
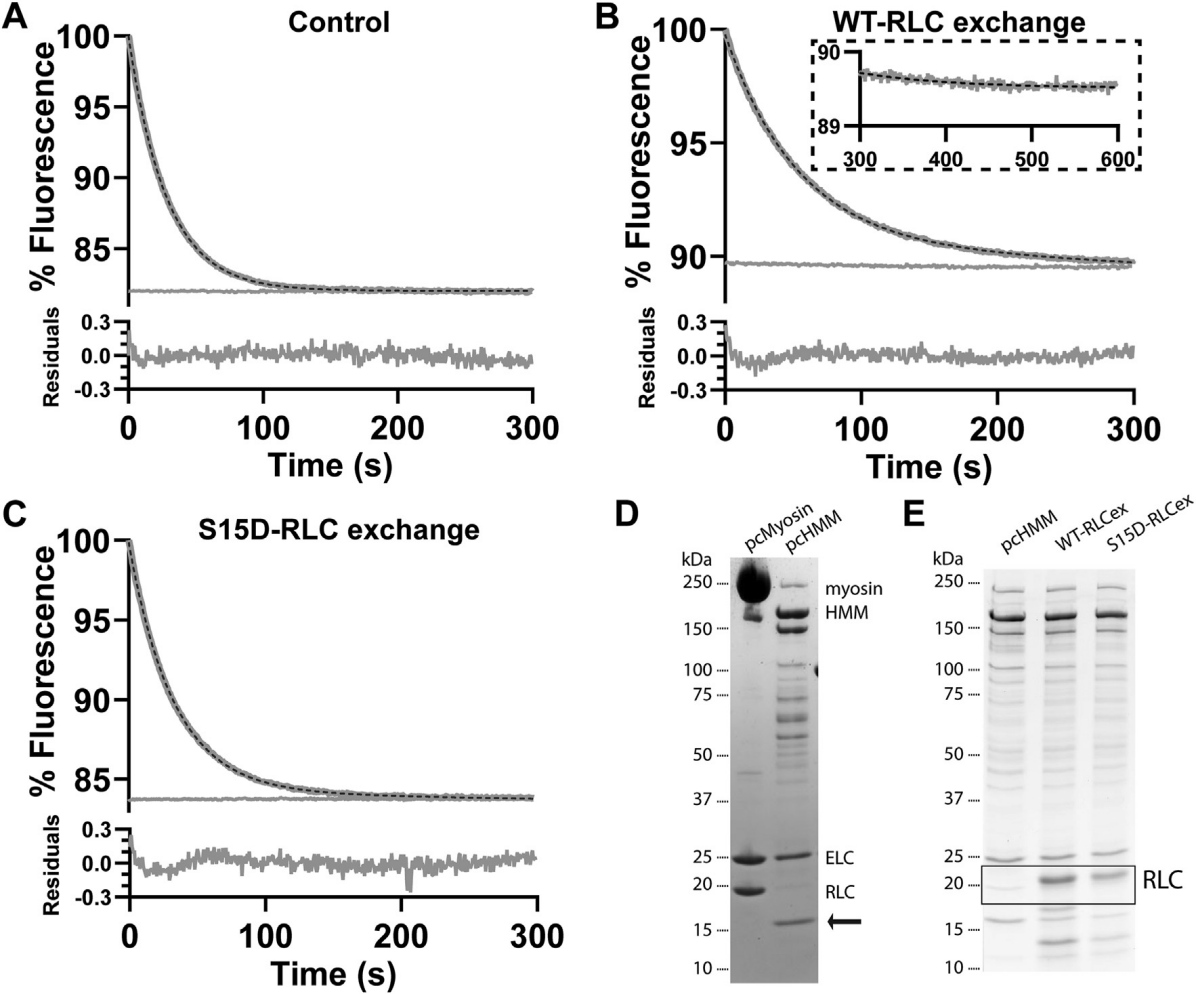
**Fluorescence changes following displacement of mantATP from pc-HMM by an excess of ATP.** In each case pc-HMM (125 nM) was preloaded with mantATP (2 μM) allowed to reach steady state (∼1 min) then rapidly mixed in the stopped flow with an excess of unlabeled ATP (250 μM). In each panel a single transient is shown over 300 s (*gray line*) with the best fit single exponential superimposed (*dashed line*). A second recording of the following 300 to 600 s is shown to establish a stable baseline at the end of the reaction. The average values of *k*_obs_ for a series of transients are listed in Table 1. The residual plot is shown below the main plot on an expanded *y*-scale. *A*, control conditions, *k*_obs_ = 0.021 s^−1^. Note that the fast noise on the data file is < ±0.1% of the total signal and the small deviation from an ideal fit to a single exponential is < ±0.3% of the total signal or < ±0.1% of the change in fluorescence. *B*, the same experiment after exchange of RLC^WT^ on HMM (inset showing expanded *y*-axis of 300–600 s), *k*_obs_ = 0.0163 s^−1^ or (*C*) RLC^S15D^ human, *k*_obs_ = 0.0181 s^−1^ RLC. *D*, SDS-PAGE of full-length pig cardiac myosin and chymotrypsin digested HMM (*arrow* pointing to clipped RLC). *E*, SDS-PAGE of HMM preexchange and post exchange of the RLC. HMM, heavy meromyosin; RLC, regulatory light chain.

Figure 2*A* presents a typical transient observed for the reaction. Note, the transient shows both data collected over the initial 300 s after mixing and the subsequent 300 s superimposed in the figure. Examining the 300 to 600 s recording on an expanded y-scale (not shown) indicated the signal change over this time to be <1% of the change over the first 300 s. The transient was therefore fitted to a single exponential with the best fit superimposed on the data (*k*_obs_ = 0.021 s^−1^). The residual plot had random noise of < ±0.1% of the total signal strength. The deviation from a flat line was < ±0.2% of the signal or <1% of the observed fluorescence change (ΔFl). The fitted single exponential can therefore account for 99% of the observed fluorescence signal change. There was no evidence of a second component larger than 1% of observed fluorescence change. Note, Figure 2*A* displays a single transient, as averaging of multiple transients gave no advantage to signal quality. Collecting repeated transient resulted in *k*_obs_ = 0.0199 ± 0.00039 s^−1^ (mean ± SEM, n = 31, all mean *k*_obs_ values are summarized in Table 1). Our analysis gives high confidence that the data can be described by a single exponential with a stable end point, as can clearly be seen from the fit line.

**Table 1:** Summary of single exponential, k_obs_ (s^−1^), values for mantATP displacement from HMM and S1

Conditions	Pc-HMM			WT-RLC			S15D-RLC			Rs-HMM			Pc-S1		
	Mean	SEM	N	Mean	SEM	N	Mean	SEM	N	Mean	SEM	N	Mean	SEM	N
Control	0.0199	0.00039	31	0.0182∗	0.00038	10	0.0181∗∗	0.00016	6	0.0334∗∗∗∗	0.0013	9	0.0155∗∗	0.00057	21
Low IS	0.0242∗∗∗∗	0.00051	14	0.0233∗	0.00064	3	-	-	-	-	-	-	0.0280∗	0.0010	12
Ca^2+^	0.0217^ns^	0.0018	16	0.0181^ns^	0.00038	6	0.0190^ns^	0.00065	5	0.02786	0.00055	6	-	-	-
Mava^a^	0.0100∗∗∗∗	0.00061	16	0.00654∗∗∗∗	0.00135	9	0.00861∗∗∗∗	0.00191	5	0.00965∗∗∗∗	0.00044	3	0.00767∗∗∗∗	0.00057	12
mant.dATP	0.0293∗∗∗∗	0.00061	12	0.0272∗∗∗∗	0.00037	12	0.0275∗∗∗∗	0.00058	5	0.0546∗∗∗∗	0.0015	6	0.0252∗∗∗∗	0.00071	6

k_obs_ for Myosin in 0.5 M KCl, k_obs_ = 0.0154 ± 0.00053 s^−1^ (N = 5).

ns, not significant; ∗*p* < 0.05; ∗∗*p* <0.01; ∗∗∗*p* < 0.001; ∗∗∗∗*p* < 0.0001 *versus* relevant control. In the top row, *p* values are compared to the pc-HMM control. In all other case the *p* values refer to the control value in the same column.

a Mava 0.5 μM for HMM, 3 μM for S1.

**Figure 3:**
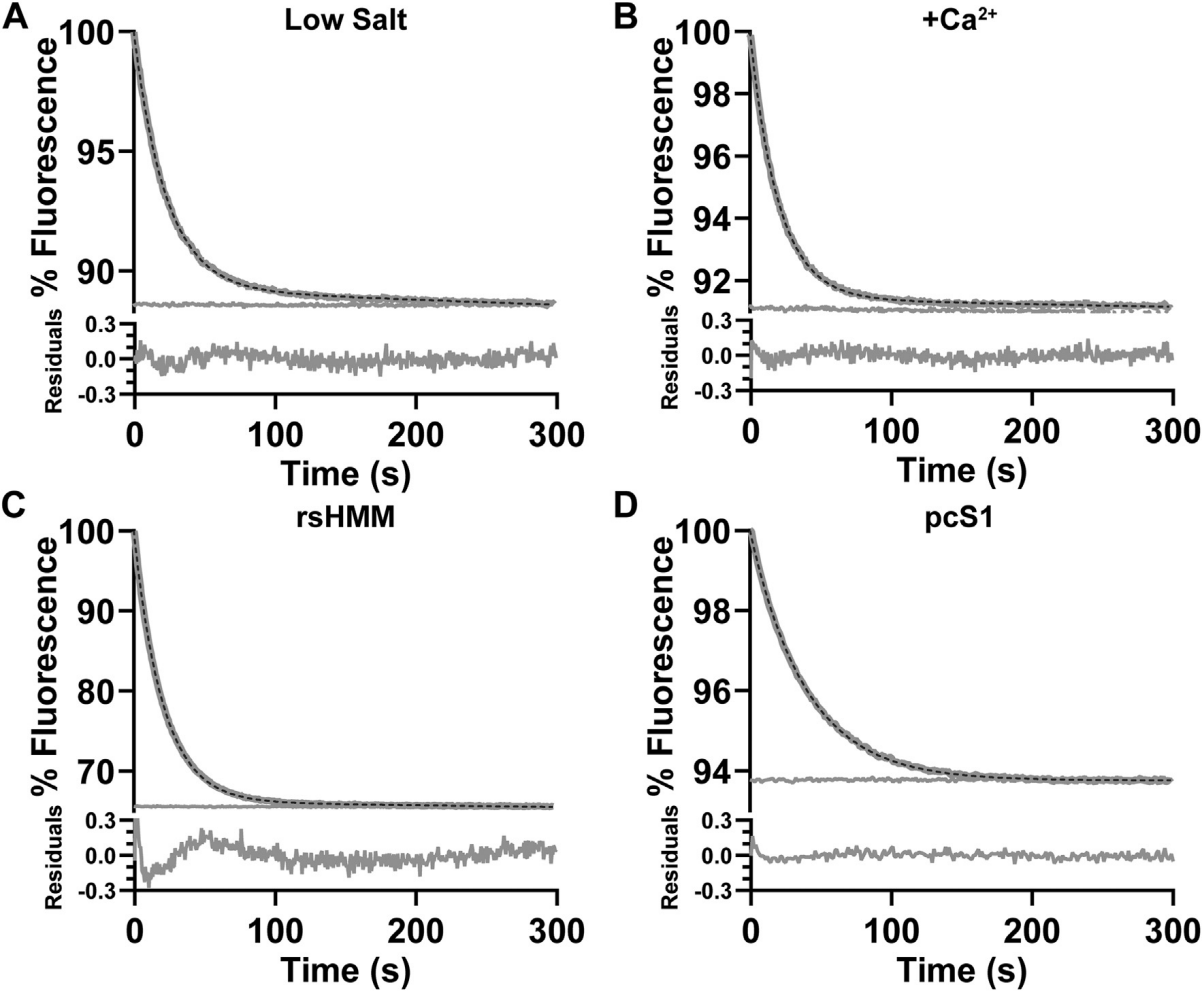
**Fluorescence changes following mant ATP displacement from pc-HMM under different conditions or with different proteins.***A*, displacement pc-HMM at low ionic strength, 20 mM KCl, *k*_obs_ = 0.0261 s^−1^ or (*B*) in the presence of excess 2 mM Ca^2+^, *k*_obs_ = 0.0270 s^−1^. *C*, displacement from rs-HMM, *k*_obs_ = 0.0282 s^−1^, and (*D*) pc-S1, *k*_obs_ = 0.0251 s^−1^. The average values of *k*_obs_ for a series of transients are listed in Table 1. HMM, heavy meromyosin; rs-HMM, rat skeletal HMM.

Based on the extensive literature of this mantATP displacement reaction, this single exponential was not expected. As such, it was important to establish the confidence with which we see a single exponential transient. We therefore undertook a series of controls measurements shown in Figures 2, B and C and 3.

### Control assays with additional expressed RLC or phosphomimetic RLC

Generating HMM by proteolysis of myosin can result in the loss or clipping of some of the RLC (Fig. 2*D*). The RLCs play a role in myosin head recruitment and are located at the hinge around which the IHM structure folds. Clipping or removing the RLC during HMM isolation could impair the ability of the HMM to form the structural IHM conformation and/or the biochemical SRX state. Similarly, phosphorylation of the RLC has been reported to reduce the presence of the structural IHM and the biochemical SRX (30, 31). We therefore replaced the native RLC present in our pc-HMM with full-length, bacterial expressed RLC (RLC^WT^) which is fully dephosphorylated (Fig. 2*B*). This control corrects for both the loss of any native RLC and ensures no potential phosphorylation that would limit the formation of the SRX state. In addition to RLC^WT^ exchanges, we also replaced the native RLC with RLC^S15D^ (Fig. 2*C*) which is reported to be a good mimic of phosphorylated RLC (32). SDS-PAGE gels of the HMM with replaced RLC are shown (Fig. 2*E*, black box | Fig. S4).

The results of mantATP displacement for both the RLC^WT^ and RLC^S15D^ exchanges (Fig. 2, *B* and *C*) demonstrated that the transients remain single exponentials with the same level of confidence as the original transient. This means that the single exponential accounts for 99% of the observed signal changes. The mean values of *k*_obs_ are summarized in Table 1, with both RLC^WT^ and RLC^S15D^ *k*obs values expressing almost a 10% (*p* < 0.044) decrease compared to controlled values. Interestingly, there was no difference between the RLC^WT^ and the phospho-mimetic RLC^S15D^ groups.

### Control assays at low ionic strength or high calcium

Modulation of myosin head interaction by ionic strength is reported to influence the ability of HMM and myosin to form the SRX state, which is stabilized at low salt (27). The assay for Figure 2*A* (100 mM KCl) was repeated at 20 mM KCl (Fig. 3*A*). The decrease in ionic strength significantly increased the mean rate constant by 20% (*k*_obs_ = 0.024 ± 0.0005 s^−1^, *p* < 0.0001) but the transient remained a single exponential decay. A similar increase in *k*_obs_ at low ionic strength was observed for pc-HMM with WT-RLC and for pc-S1 (Table 1 and Fig. S2).

The essential light chain is also known to bind calcium, and this could influence the formation of the SRX state (33). The transient of Figure 2*A* was repeated in the presence of high Ca^2+^ (2 mM Ca^2+^EGTA, free [Ca^2+^] ∼30 μM) (Fig. 3*B*). A single exponential with a mean *k*_obs_ = 0.0217 ± 0.0018 s^−1^ was not significantly different from control values. A similar lack of effect of Ca^2+^ was observed for rat skeletal HMM (rs-HMM), and pc-HMM with exchanged RLC^WT^ and RLC^S15D^ (Table 1 and Fig. S2).

**Supporting Figure S1:**
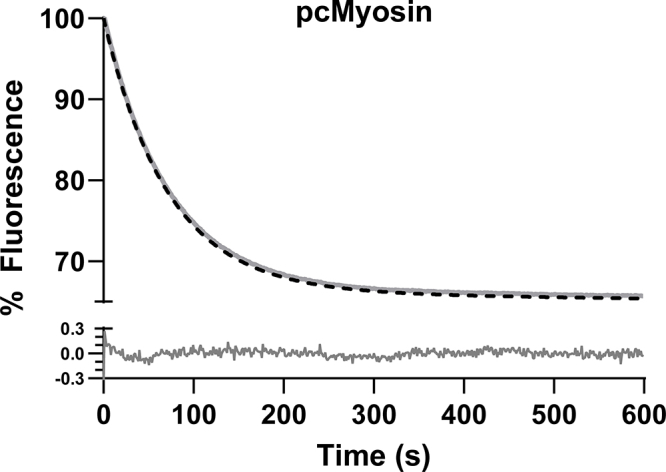


**Supporting Figure S2:**
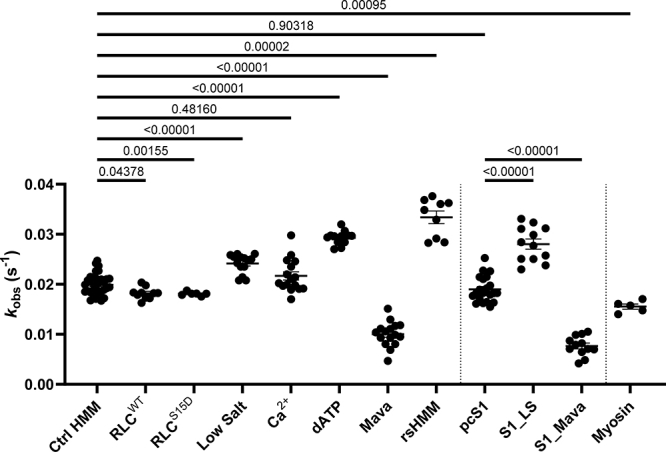


To test whether the HMM results were due to a specific issue with our pc-HMM, we tested myosin and its subfragments from different sources including HMM from rat back muscle (rs-HMM), porcine cardiac ventricle subfragment 1 (pc-S1) and full length porcine cardiac myosin. In all cases, we observed fluorescence transients that were well described by a single exponential. The data from rs-HMM and pc-S1 are displayed in Figure 3, *C* and *D* and the full-length myosin in Fig. S1. The quality of the data remains the same, with no evidence of a slow phase in the reaction. The mean values of *k*_obs_ are summarized in Table 1 and Fig. S2.

### Mavacamten inhibits while deoxy-ATP accelerates the single exponential displacement reaction

**Figure 4:**
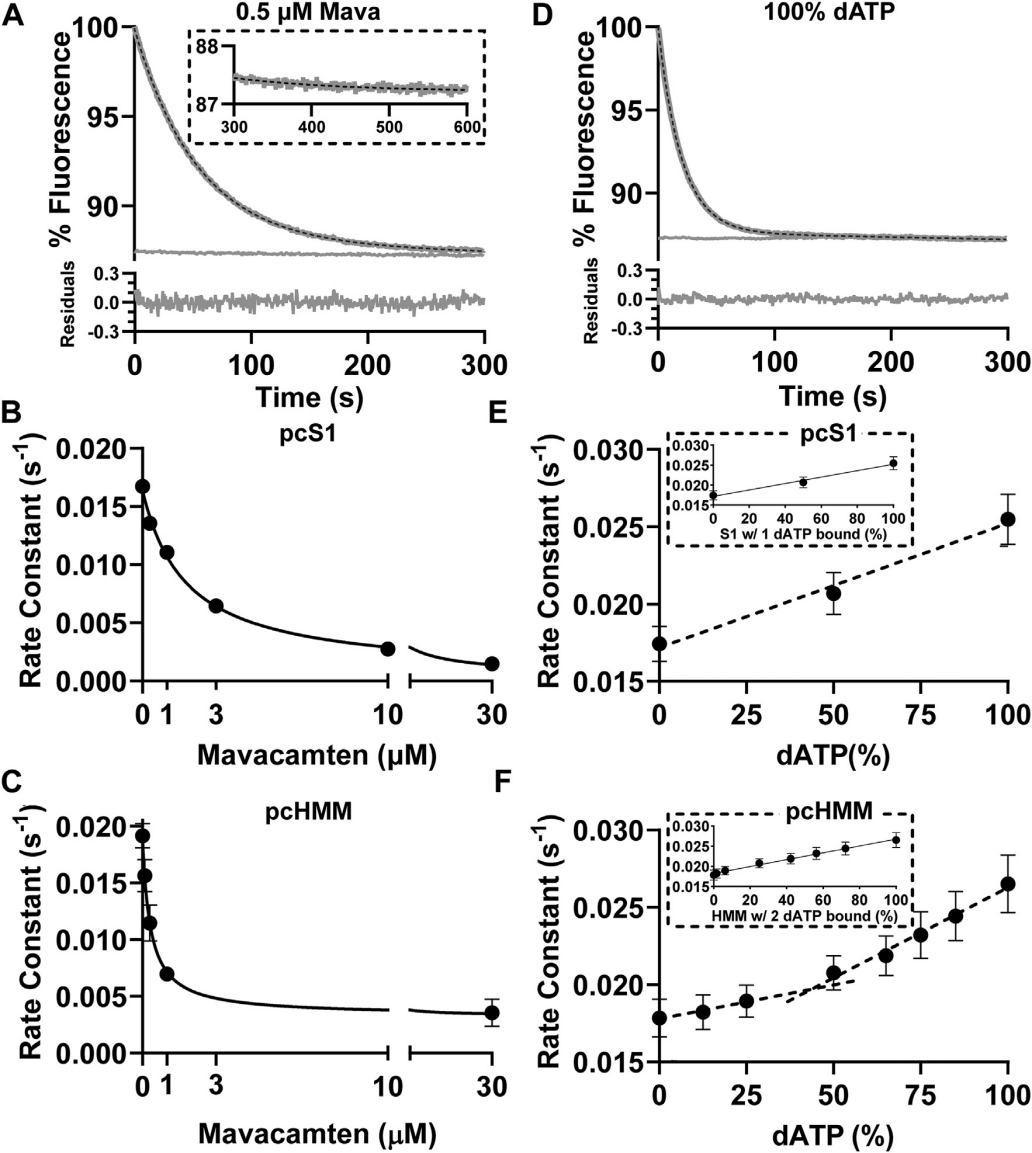
**Fluorescent changes following mant.ATP displacement from pc-HMM and pc-S1 with added mavacamten or replacing mant.ATP with mant-dATP**. *A*–*C*, Mava. *D*–*F*, percentage of the mant.ATP replaced by mant.dATP. The data for (*A* and *D*) are shown in the same format as in Figure 2. *A*, with pc-HMM and 0.5 µM Mava (inset showing expanded *y*-axis of 300–600 s), (*D*) with mant.dATP replacing mantATP. Titrations of *k*_obs_ for pc-S1 (*B* and *E*) or pc-HMM (*C* and *F*) with Mava (*B* and *C*) or mant.dATP (*E* and *F*). Titrations of *k*_obs_ with Mava are fitted to a binding isotherm and titrations of mant.dATP fitted to straight lines. HMM, heavy meromyosin; pc-HMM, porcine cardiac HMM.

As mentioned earlier, Mava has been widely utilized as a myosin inhibitor (3, 20, 34, 35). One interpretation of how Mava inhibits myosin is by stabilizing the prepower stroke M.ADP.Pi form of myosin which leads to stabilization of the SRX state and ordering of IHM myosin heads on the thick filament. In Figure 4*A*, the effect of 0.5 μM Mava on the mantATP displacement reaction was examined. The presence of 0.5 μM Mava inhibited the turnover of mantATP and slowed *k*_obs_ from control values by a factor of 2 (from 0.0199 ± 0.0004 to 0.0100 ± 0.00061 s^−1^, *p* < 0.0001). This inhibited decay, however, remained a single exponential. For completeness, we treated pc-S1 with 3 μM Mava. Our results still expressed a single exponential decay. The value of *k*obs was reduced by a factor of ∼2 (0.0155 ± 0.00057 s^−1^ to 0.00767 ± 0.00057 s^−1^, *p* < 0.0001 see Table 1). Inhibition of a single exponential process was also seen for pc-HMM with replaced RLC (2-3-fold) and for rs-HMM (3-fold, Table 1 and Fig. S2).

To confirm the K_i_ values for Mava, the pc-HMM and pc-S1 assays were repeated over a range of Mava concentrations from 0 to 30 μM (Fig. 4, *B* and *C*). In all cases, a single exponential was produced with best fit to a *k*obs titration curves, yielding Ki of 1.76 μM for S1 and 0.32 μM for HMM.

To complete our set of displacement assays, we also utilized the myosin activator mant-labeled 2′ deoxyATP (mant.dATP) to investigate the nucleotide displacement reaction. Both dATP and mant.dATP, are reported to accelerate myosin ATPase activity, disrupt the ordered thick filament structure, and destabilize the SRX through alterations in the nucleotide binding pocket (17, 36, 37). This disruption results in increased charge on the actin binding surface of myosin (38) results in elevation of the myosin heads from the thick filament backbone (26, 36, 39). Figure 4*D* illustrates that replacing mantATP with mant.dATP in the assay results in an accelerated single exponential displacement. The mean value for *k*_obs_ increased by ∼50% for both pc-HMM (to 0.029 ± 0.0006 s^−1^, *p* < 0.0001) and for pc-S1 (to 0.025 ± 0.0007 s^−1^, *p* < 0.0001), as listed in Table 1. Titrating pc-HMM with mant.dATP from 0% to 100% resulted in a biphasic increase in *k*obs with an apparent break point at ∼50% mant.dATP (Fig. 4*F*). This may indicate a different behavior of pc-HMM with mant.dATP occupying one head compared to two. Replotting the data as *k*obs *versus* the fractional occupancy of HMM with both heads binding mant.dATP generates a linear relationship (Fig. 4*F*, insert). Repeating the titration with mant.dATP for pc-S1 again only resulted in single exponential decays, with a similar 50% increase in *k*obs (Fig. 4*E*). The *k*obs was linearly dependent upon the fractional occupancy of S1 with mant.dATP.

The remarkable conclusion from these results is that we can find no evidence of a secondary slow phase in any of the assays performed. We conclude that the mantATP displacement assay, as used widely in the literature for estimating the fraction of myosin in the SRX state, is not reliable when used with purified proteins. This finding does not mean that there is no SRX state, but that the mantATP displacement assay cannot reliably quantify the population of the SRX state. In additions to our experimental findings with HMM presented here, there are good theoretical arguments for being cautious about the interpretation of the assay when investigating myofibrils or muscle fibers as described in the introduction.

### Single and multiple turnover assays with mantATP

**Figure 5:**
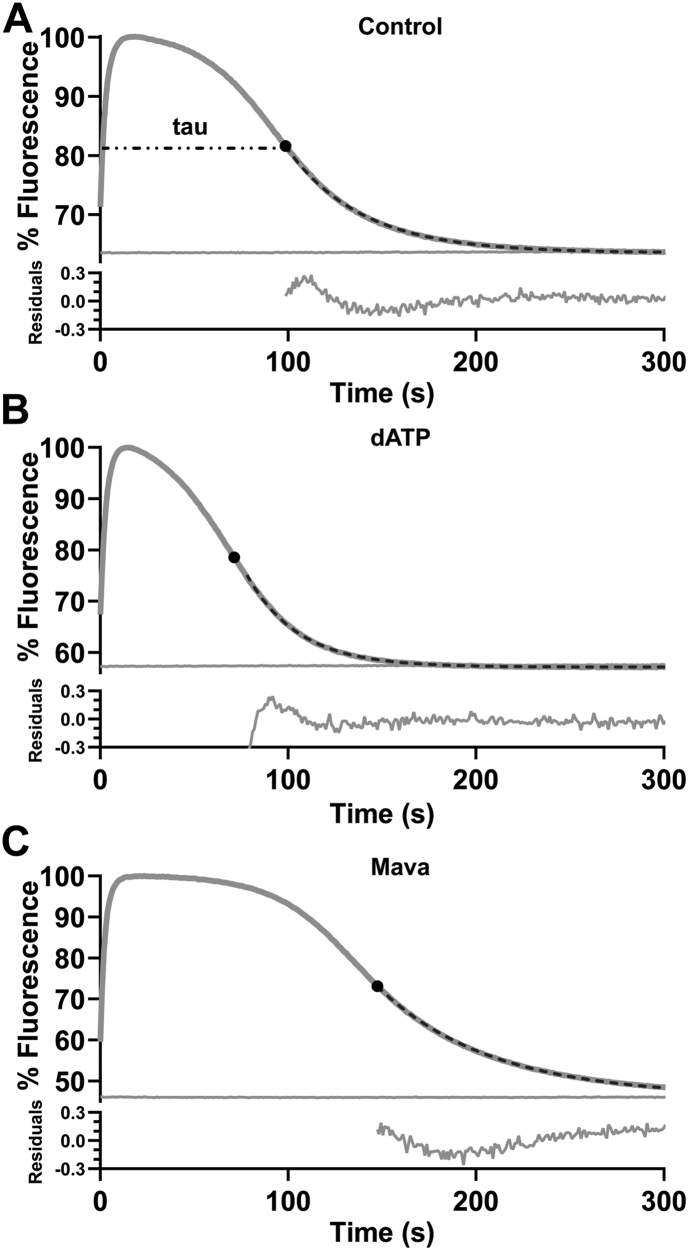
**Measuring the basal mantATP turnover rate (*k***_**cat**_**) using a multiple turnover assay in the stopped flow.***A*, turnover of mantATP (0.5 μM) by pc-HMM (0.25 μM) followed using excitation at 297 nm and emission at λ > 400 nm. Tau (τ) the time to turnover the mantATP is estimated as duration until the fluorescence increase returns to 50% of its starting value. A single exponential (*k*_obs_) is also shown fitted to the final 50% of the transient. *B*, Same as (*A*) but with mant.dATP replacing mantATP. *C*, Same as (*A*) but in the presence of 0.5 μM Mava. HMM, heavy meromyosin; pc-HMM, porcine cardiac HMM.

One of the limitations of much of the literature on the mantATP displacement assays is that a control steady-state ATPase is not presented alongside the mantATP displacement assays. Where they are presented, the two rarely agree (3, 18, 29). Furthermore, the effect of Mava on the two assays has different sensitivities (29). To assess *k*_cat_ in our mantATP displacement assays, we performed a multiturnover experiment where the fluorescence was monitored as pc-HMM turned over a small excess of mantATP (Fig. 5). In this case, 250 nM pc-HMM was mixed with a 2-fold excess of mantATP. The fluorescence was observed to increase to a steady-state value and then decayed back to the original signal level (Fig. 5*A*). The addition of a small amount of actin (0.3 μM) to the reaction ensures mantADP is displaced at the end of the reaction to attain the starting fluorescence value. This low actin concentration at 100 mM KCl has an insignificant effect on the turnover rate. The length of time tau (τ) until the fluorescence has returned to 50% of the peak value gives an estimate of the time taken to hydrolyse the mantATP. From this, *k*_cat_ = [mantATP]/([HMM] ∗ τ).

**Supporting Figure S3:**
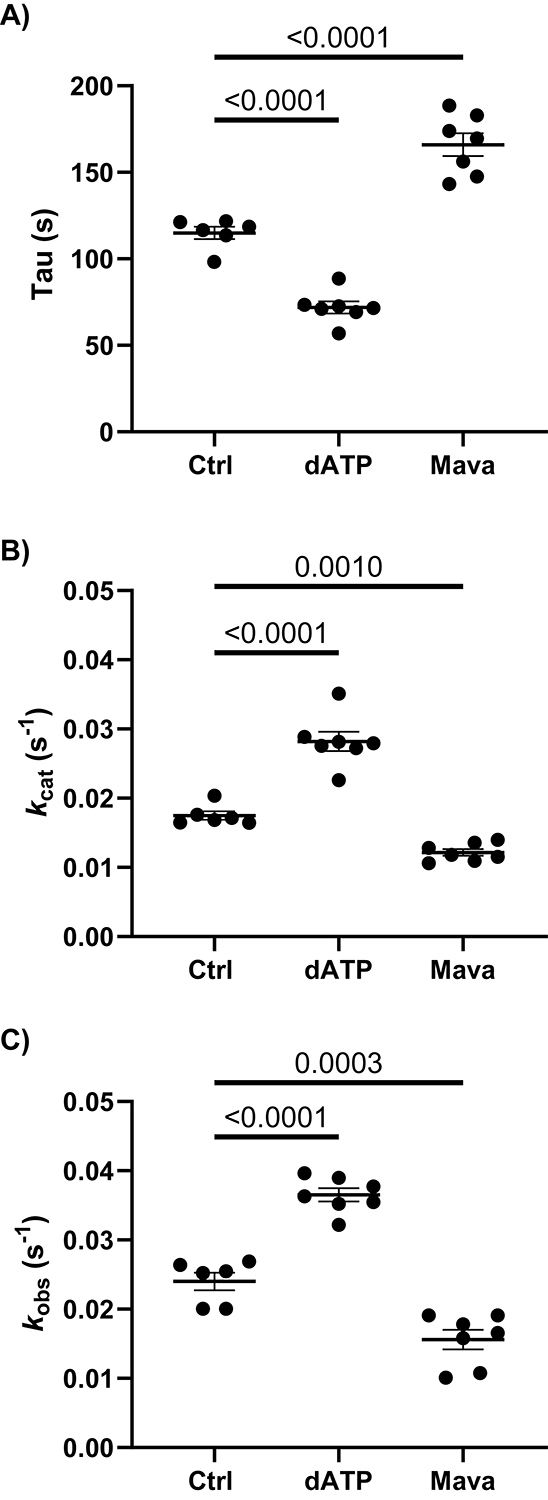


**Supporting Figure S4:**
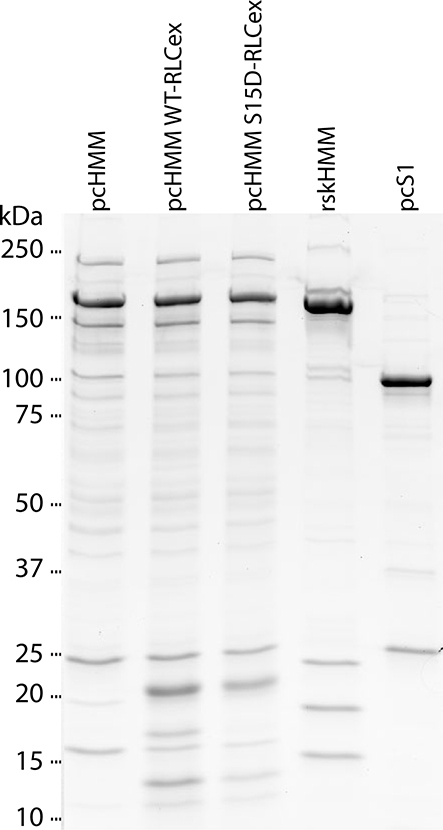


Adding 0.5 μM Mava to the assay increased the length of the steady-state (Fig. 5*C*) and replacing mantATP with mant.dATP shortened the steady-state (Fig. 5*B*). This provides estimates of *k*_cat_, for the control, Mava, and mant.dATP of 0.0175 ± 0.0006, 0.0122 ± 0.0004, and 0.0282 ± 0.0014 s^−1^, respectively (Table 2 and Fig. S3). These values, which depend upon an accurate measure of the active protein concentration, are within 30% of the values of *k*_obs_ from the mantATP displacement assay listed in Table 1.

**Table 2:** Summary of multiple turnover tau (s), k_cat_ (s^−1^), and k_obs_ (s^−1^) values for mantATP displacement from pc-HMM

Conditions	Tau (s)			k_cat_ (s^−1^)			k_obs_ (s^−1^)		
	Mean	SEM	N	Mean	SEM	N	Mean	SEM	N
Control	115	3.569	6	0.01749	0.00060	6	0.02402	0.00127	6
mant.dATP	71.93∗∗∗∗	3.485	7	0.0282∗∗∗	0.00139	7	0.03651∗∗∗	0.00096	7
Mava	166∗∗∗∗	6.581	7	0.01217∗∗∗∗	0.00049	7	0.01561∗∗∗∗	0.00142	7

*p* values represent ∗*p* < 0.05; ∗∗*p* < 0.01; ∗∗∗*p* < 0.001; ∗∗∗∗*p* < 0.0001 *versus* control within each column.

## Discussion

We have presented a series of assays using mantATP displacement by ATP to evaluate the turnover of mantATP by HMM. As described in our Results, we find no evidence of two components in the reaction. All measurements resulted in a clean single exponential decay with *k*_obs_ values that match the expectations from steady state ATPase assays. Single exponential displacement reactions were seen for single headed S1 (where no IHM is expected) and for two headed HMMs where the formation of an IHM is possible and has been reported in structural studies of isolated HMM, myosin molecules, and sarcomere thick filaments.

### Why does the literature contain many reports of double exponential fits to the displacement data, defining the SRX and DRX populations for mammalian cardiac myosin?

Our present studies are limited to HMM and S1 in solution, so we cannot usefully comment on the data from myofibrils, skinned muscle fibers, whole myocytes, or intact myocytes. Each preparation has its own experimental complexities and contains many additional protein components that can affect the IHM and/or SRX (IHM/SRX) states such as the thick filament backbone, titin, and myosin binding protein-C. In each experimental preparation, however, the set of paradoxes outlined in the Introduction remain. The most significant is whether the myosin heads in the IHM/SRX states are in equilibrium with liberated/DRX states. If they are in equilibrium, then the myosin must be interchanging on a time scale like or slower than the ATP turnover of heads in the SRX state of myosin (>100 s). This is incompatible with measured rates of formation and breakdown of the SRX state in the mantATP assay.

Apart from original data from the Cooke laboratory (utilizing muscle fibers) (2, 40), too few studies present detailed controls to evaluate any potential problems with experimental fitting. In many cases, only the percent SRX (%) is reported. The biexponential fitting of the data requires careful evaluation of the fitting procedure to precisely define two components. Some issues in defining two components were previously discussed by Walklate *et al.* and Ma *et al.* (17, 26). The stability of the baseline and the extent of photobleaching over the extended time of the reaction can seriously affect the quantification of the slow component. Similarly, missing information over the first few seconds of the transient can distort the evaluation of the fast component. Distortion of either the fast or slow phases then has consequences for how the best-fit evaluates the contribution of each component. Without controls, it is difficult to assess any underlying problem in the data. In addition, it is essential to evaluate the basal turnover reaction by steady state ATPase assays alongside mantATP displacement assay. The two should be in reasonable agreement. If they do not agree, then there is a problem somewhere either in the data or the interpretation.

Few studies of the mantATP displacement reaction have been reported for the HMM reaction with most utilizing myofibrils, muscle fibers, or myocytes. Exceptions are the work of the Thomas and Muretta groups (19) with bovine cardiac HMM and a human β-cardiac myosin fragment expressed in mouse C2C12 cells (3). The bovine HMM studies used a FRET pair of tags on the RLCs to distinguish two populations of HMM, one with a short distance between the RLCs assumed to reflect the IHM structure, which was 43% of the tagged population (27). Curiously, low ionic strength or addition of Mava did not significantly increase the population of IHM present, even though in the parallel measurements using the mantATP displacement assay they did see an increase in the populations of SRX. Chu *et al.* (27) interpreted the data to mean that the SRX and IHM were not the same and two assays measured different states of HMM.

The human HMM construct used by Anderson *et al.* is not identical to the HMM produced by proteolytic cleavage of tissue purified myosin. The HMM construct was expressed in mouse C2C12 cells and consisted of the myosin motor and light chain binding domains followed by 15 or 25 of the heptad repeats which make up the proximal tail. The construct also contained, at the C terminus, a leucine zipper to ensure correct assembly of the dimer, a GFP, and a small peptide at the C terminus to facilitate surface attachment *via* flexible linkers (3). This construct does show two phases for the mantATP displacement reaction and the fraction of slow SRX phase increases with Mava and decreases with several HCM linked mutations. Similar results were reported for the human S1 construct. However, careful analysis of the published data shows discrepancies between the measured basal ATPase activity, its inhibition by Mava, and the values predicted based on the mantATP displacement data. The predicted Mava inhibited *k*_cat_ value, based on their estimates of % SRX and the *k*_obs_ for mantATP displacement is five times higher than the measured *k*_cat_ (Table S1). This suggests that differences in our results compared with others may be due to differences in experimental design or in the protein construct used. This could be resolved by parallel studies of the two proteins.

### Does a single exponential fit to the displacement data mean there is no IHM or SRX in our HMM assays?

Our results do not mean that there is no structural IHM and/or biochemical SRX for HMM in solution. They only demonstrate that this mantATP assay is not reliable for evaluating the fraction of myosin in the IHM/SRX states in solution. It is technically difficult to distinguish if a decrease in *k*_obs,_ caused by an inhibitor such as Mava, is a result of impaired Pi release and/or increases in the IHM/SRX states. Furthermore, if the IHM/SRX states are present, they should theoretically only slow down the *k*_obs_ and not generate a second phase. A double exponential displacement reaction would be observed if the biochemical interconversion of the SRX and DRX states are much slower than the turnover. The scenario with only a single phase was observed for the calcium-regulated scallop HMM, where the ADP release is slowed from 15 s^−1^ in the presence of calcium (*On-state*) to <0.5 s^−1^ in its absence (*Off-state*) (41). Evidence of a folded back structure for scallop HMM in the absence of calcium, and similar to the IHM, was provided by both negative stain EM and ultracentrifugation analysis (10, 42, 43).

The presence of Ca^2+^ and/or the phosphomimic RLC^S1^5D mutation, resulted in little effect on the *k*_obs_ values reported for HMM in Table 1. This is consistent with no inhibition of the rate of ATP turnover due to the occupancy of IHM/SRX conformations. This contrasts with studies of thick filaments in muscle fibers where both treatments (presence of Ca^2+^ and RLC phosphorylation) increase the release of heads from the ordered conformation on the filament backbone (44, 45). It is important to note that these observations in thick filaments are compatible if the IHM/SRX states require additional external factors present along the thick filament for stability. Regarding purified protein assays, if there is little IHM/SRX in isolated HMM, then its presence will not be reduced significantly by Ca^2+^or phosphorylation.

The data presented here provide no firm evidence of any SRX state as originally defined. The observation that Mava inhibits HMM and S1 to a similar extent would argue that this is primarily an inhibition of Pi release with little induction of SRX in the case of HMM. The similar levels of activation of S1 and HMM by dATP also argue that there is little loss of any SRX state in HMM in the presence of dATP. Instead, there is evidence of cooperativity between the two heads in binding Mava or dATP. Figure 4, *E* and *F* show some evidence of cooperative behavior in the effect of mant.dATP on the displacement reaction. For pc-HMM, the effect of percent mant.dATP present on *k*_obs_ is clearly biphasic with a larger effect above 50% mant.dATP than below. This effect is absent from the S1 data, although the degree of acceleration of *k*_obs_ is similar. Replotting the HMM data as the fraction of HMM with both heads occupied by dATP† is linear, suggesting both heads need to bind mant.dATP to produce the full effect on *k*_obs_.

The data also show evidence for cooperative Mava binding between the two heads of HMM. Mava inhibits the acto-S1 ATPase reaction quite strongly, as originally reported with a K_i_ of ∼0.2 to 0.5 μM (19, 46). Few studies have reported the K_i_ of Mava for S1 or HMM to assess the relevant ATPase for stabilizing the M.ADP.Pi conformation of myosin under relaxing conditions. The K_i_ values for pc-S1 (1.76 μM) and pc-HMM (0.37 μM) are quite distinct, and clearly show a stronger affinity of Mava for pc-HMM. This is consistent with some form of positive cooperativity between the two heads and the binding of Mava, with the binding of one Mava to HMM increasing the affinity of binding of a second Mava. Cooperative binding of Mava or dATP to HMM and not S1 suggest a structural communication, independent of IHM, between the two heads even though we see no difference in the effects of Mava or dATP at saturation on the ATP turnover rates of S1 and HMM.

The implications for our results and interpretations for the clinical use of Mava are relatively benign. Mava remains an inhibitor of actomyosin ATPase as reported in the original work (46). Whether the mechanism of inhibition is through stabilization of the prepower stroke conformation (M.ADP.Pi) through the inhibition of Pi release, the structural sequestration of myosin heads (IHM), and/or the ordered stabilization of the thick filament makes no difference to its use. Due to mass action, any factor which stabilizes the M.ADP.Pi conformation is also likely to increase the probability of IHM formation if both heads are trapped in the M.ADP.Pi form.

### Concluding remarks

The ease of making the mantATP SRX measurements has led to many studies of this type for a variety of muscle tissues samples. The difficulty in making accurate assignment of the two phases has led to a series of paradoxes and confusion over what exactly has been measured. This confusion is made worse when trying to correlate the biochemical measurements of ATP turnover with structural assays of the IHM and the order-disorder transition of thick filaments. We have shown here that careful measurements with HMM eliminate the apparent paradoxes and leads to a self-consistent model for how mantATP, mant.dATP and Mava interact with the two heads of HMM. The mantATP turnover assay is not suitable for defining the difference between a biochemically inhibited myosin head and the IHM, where present. This requires a more structural based approach such as sedimentation, as used for solutions of smooth and scallop HMM, although detailed interpretation is challenging unless the protein is homogeneous. Cryo-EM of HMM is also possible but, again, precise counting of IHM *versus* disordered heads is challenging. The use of fluorescence lifetimes with FRET and the single molecule imaging of fluorescent ATP (27) in myofibrils both have the potential to aid the resolution of the remaining questions. At this stage, the important message is to be careful in defining what is measured in each assay and not to use terms such as IHM and SRX without careful definition.

## Experimental procedures

### Animal use and ethics

All experiments followed protocols approved by the University of Washington Institutional Animal Care and Use Committees according to the “Guide for the Care and Use of Laboratory Animals” (National Research Council, 2011). Sprague-Dawley rats aged 8 to 10 weeks were euthanized following approved protocols prior to muscle dissection and HMM isolation. Farm bovine and porcine hearts were obtained immediately after the animal was euthanized and rinsed in cold oxygenated Tyrode’s buffer.

### Protein preparations

Striated cardiac and skeletal muscle heavy meromyosin was isolated as previously described (47). In brief, porcine left cardiac ventricular muscle, and rat back muscles (longissimus and iliocostalis lumborum) were roughly minced on ice. Minced tissue was then added to extraction buffer (0.3 mM KCl, 0.15 mM imidazole, 10 mM Na_2_P_2_O_7_, 1 mM MgCl_2_, 2 mM DTT, pH 6.8) and stirred on ice for approximately 30 min in a small beaker. Excess muscle residue and actin filaments were then removed by centrifugation at 260,000*g* for 1 h. The supernatant was then diluted 10- to 15-fold with 4 °C water containing 2 mM DTT and left on ice for 1.5 h to allow for myosin precipitation. Samples were then spun at 44,000*g* to pellet precipitated myosin. Myosin was then dissolved in a high salt solution (0.6 M KCl, 2 mM MgCl_2_, 2 mM DTT, 10 mM, pH 7.0).

For pc-HMM, dissolved myosin was digested by chymotrypsin (tosyl-L-lysyl-chloromethane hydrochloride treated, Sigma-Aldrich C3142) (50 μg/ml) at 25 °C for 10 min. For separate preparations of rs-HMM, dissolved myosin was digested by chymotrypsin (25 μg/ml) at 25 °C for 10 min. Each digestion was stopped by adding 4-fold of PMSF solution (2 mM MgCl2, 5 mM EGTA, 5 mM DTT, 10 mM Imidazole, 0.2 mM PMSF, pH 7.4) and left on ice for 1 h. Precipitated light meromyosin was removed by spinning at 45,000*g* for 20 min. Striated HMM was stored at −80 °C with the addition of 1% sucrose and 1% protease inhibitor (Sigma-Aldrich). *Note, throughout the article, all concentrations of HMM refer to the concentration of heads.*

Porcine cardiac sub-fragment one (pc-S1) was isolated as previously described (48, 49). Porcine cardiac myosin was dissolved in a salt solution (120 mM NaCl, 12.3 mM NaH_2_PO_4_, 7.7 mM Na_2_HPO_4_, 1 mM EDTA, pH 7.0) to achieve a myosin concentration of 10 mg/ml. Once dissolved, chymotrypsin was added to a 30 μg/ml final concentration. The myosin was digested for 15 min before being stopping with PMSF (0.4 mM final concentration in solution), in a solution of 0.1 mM NaCO_3_, 0.1 mM EGTA, 1 mM DTT, pH 7.0, and 6 mM MgCl_2_. The protein was centrifuged at 620,000*g* for 20 min to isolate the pc-S1 from the residual precipitate light meromyosin and undigested myosin and then stored at −80 °C with the addition of 1% sucrose and 1% protease inhibitor.

Actin filaments for multiturnover assessment were generated as previously described (50). Briefly, bovine cardiac G-actin was incubated in polymerization buffer (50 mM KCl, 2 mM MgCl_2_, and 1 mM ATP) and allowed to assemble for 2 h at 4 °C. The polymerized actin was then dialyzed against 500× volume of working buffer (50 mM Tris–HCl, 100 mM KCl, 5 mM MgCl_2_, 1 mM EDTA, pH 7.2 at 4 °C) overnight. The next day, the actin was stabilized with unlabeled phalloidin by adding a 1:1 molarity concentration of phalloidin to actin and incubated overnight at 4 °C. Actin was stored at 4 °C for up to 3 weeks.

### Recombinant regulatory light chain production

The human cardiac RLC in pET3d vector was originally gifted by Dr Szczesna-Cordary’s lab at the University of Miami Health System. Further subcloning into pET24a expressing vector with the addition of a flag-tag at its C terminal was done following the standard molecular biological techniques. Site-directed mutagenesis was performed using the QuikChange II Site-Directed Mutagenesis Kit (Stratagene) to substitute Serine 15 in RLC (WT) with aspartic acid (S15D). The DNA sequences of these two expressing constructs were verified by DNA sequencing. The expression of the recombinant proteins in *Escherichia coli* (BL21) was done following the protocols previously developed in our lab (51). The expressed recombinant proteins were extracted from bacterial cells and purified on diethylaminoethanol Fast column equilibrated by 6 M urea, 25 mM Tris at pH 8.0, 1 mM EDTA and 1 mM DTT. Proteins were eluted with a salt gradient washing in the same buffer from 0 to 0.25 M NaCl. The fractions containing the desired purified protein and their concentrations were monitored by SDS PAGE and DU 800 Spectrophotometer. Protein purity was >95% as assessed by SDS-PAGE with Coomassie stain. Protein concentration was calculated by spectrofluorometer absorbance at 280 nm and adjusted with the extinction coefficient. The proteins were aliquoted and saved in −20 °C freezer before use.

### Regulatory light chain exchange

Recombinant RLC^WT^ and RLC^S15D^ was exchanged into pc-HMM as previously described (52, 53). Briefly, both pc-HMM and recombinant RLC were dialyzed overnight against exchange buffer (50 mM Hepes, 500 mM NaCl, 10 mM EDTA, 10 mM DTT, pH 7.6). The molarity of pc-HMM (per head ∼170 kDa) and RLC were calculated and then combined in exchange buffer at a 10-fold molar excess of RLC to pc-HMM and incubated at 30 °C for 30 min. The reaction was stopped by adding 12 mM final concentration of MgCl_2_ and placed on ice for 30 min. Excess RLC protein was removed by dialyzing overnight against wash buffer (5 mM NaH_2_PO_4_, 10 mM sodium acetate, 4 mM magnesium acetate, 2 mM DTT, pH 7.0). Exchanged pc-HMM-RLC was stored at 4 °C and used within 2 days.

### Kinetic assays

All kinetic assays were performed using a HiTech Scientific Stopped Flow system equipped with a Hg/Xe lamp and monochromator. Experiments were performed in rigor buffer (0.1 M KCl, 50 mM Tris, 2 mM MgCl_2_, 1 mM EGTA, pH 7.1 at 21 °C). KCl was adjusted to 0.02 M for low salt experiments and 0.5 M KCl for full length myosin experiments to provide predominantly monomeric myosin. Tryptophan fluorescence at 297 nm was utilized as a FRET fluorescence to excite mantATP (Jena Bioscience), which was observed at above 400 nm at 90° to the incident light through a KV400 optical filter. This technique was utilized instead of direct 365 nm excitation of mantATP due to the large excess of mantATP:protein (16:1) which resulted in a significant fluorescence bleaching over the collected measurement time scale. This means that all measurements only monitor mantATP bound to the protein and not the large excess mantATP free in solution. To correct for lamp variability and drift over the >3 min observation period, a beam splitter was introduced into the exciting light path and a small fraction of the light was recorded as a lamp reference channel in parallel with the fluorescence signal. This dual light beam recording setup provided a reference signal of lamp stability, and the fluorescence signal was normalized against the reference channel. Data were stored as 500 time points for each transient and each point represents the average of 48 individual reading of the photomultiplier output. This combination of conditions resulted in fluorescent signals with very low signal noise over the complete time course along with very stable baselines and minimized photobleaching. For each transient, the data were collected on a 300 s time base to allow accurate definition of the transient, followed by a further 300 s recording to allow accurate definition of the baseline signal. In all cases the base line was flat and stable over 300 to 600 s. The low noise on each transient meant that there was no advantage in averaging individual transients as is the normal practice. Instead, each transient was analyzed individually and the *k*_obs_ values averaged and reported in Table 1 as the mean ± SEM of transients as listed.

### Data analysis

Data were analyzed using the software supplied with the equipment. This provides a least-squares best fit of the data with a range of functions including a single exponential (Fl_t_ = Fl_0._exp^−kobs.t^ + baseline), as used here. In addition, a residual plot quantifying the difference between the observed signal and the fitted function is provided to evaluate the quality of the fitted function. In all cases the fitted line can describe >99% of the signal change observed.

Statistical analysis of mean k_obs_ values used one-way analysis of variance (ANOVA) with Dunnett’s multiple comparison. Normality was confirmed using the Kolmogorov–Smirnov test.

## Data availability

All data are stored on a cloud SharePoint platform and access is available upon request. Please inquiry with corresponding author at mregnier@uw.edu.

## Supporting information

This article contains supporting information.

## Conflict of interest

The authors declare that they have no conflicts of interest with the contents of this article.

## Footnotes

† If the fraction of total mantATP present as mant.dATP is α, and mantATP and mant.dATP bind a head with similar affinities then the fraction of HMM present with both heads binding mant.dATP is α^2^.
