# Supplementary material for: The biochemically defined super relaxed state of myosin—A paradox
Source: J Biol Chem. 2023 Dec 14;300(1):105565. doi: 10.1016/j.jbc.2023.105565 (PMC10819765; doi:10.1016/j.jbc.2023.105565)
Supplement: Supporting Table S1 [file mmc1.docx]

**Table S1: Analysis of Anderson et al (3) ATPase data** at 100 mM Kac. Data from Supp Information Figure S2 & S5. k_cat_ predicted is calculated as %SRX.k_SRX_/100 + (100-%SRX).k_DRX_/100

|  | **Mava 10 µM** | **%SRX** | **k_SRX_ (s^-1^)** | **k_DRX_ (s^-1^)_** | **k_cat_ measured (s^-1^)** | **k_cat_ predicted (s^-1^)** | **predicted/obs** |
| --- | --- | --- | --- | --- | --- | --- | --- |
| **25-hep HMM** | - | 25 | 0.0034 | 0.03 | 0.019 | 0.02335 | 1.228947 |
|  | + | 90 | 0.0027 | **0.02/0.03*** | 0.001 | 0.00443 | **4.43/5.43** |
| **2-hep HMM** | - | 7 | 0.0018 | 0.022 | 0.026 | 0.020586 | 0.791769 |
|  | + | 60 | 0.0018 | 0.019 | 0.0017 | 0.00868 | **5.105882** |
| **S1** | - | 12 | 0.0013 | 0.021 | 0.0123 | 0.018636 | 1.515122 |
|  | + | 52 | 0.0013 | 0.013 | 0.0014 | 0.006916 | **4.94** |

* There is no estimate of k_DRX_ for 25-hep HMM + Mava since there is little DRX present. I have assumed either a zero or 50% inhibition of k_DRX_ 50% is similar to that for S1. This does not affect the conclusion of a 4-5 fold discrepancy between predicted and measures k_cat_.
