# Supplementary material for: The biochemically defined super relaxed state of myosin—A paradox
Source: J Biol Chem. 2023 Dec 14;300(1):105565. doi: 10.1016/j.jbc.2023.105565 (PMC10819765; doi:10.1016/j.jbc.2023.105565)
Supplement: Supporting Figures S1–S4 legends [file mmc2.docx]

Supporting Information:

Figure Legends:

Figure S1. Fluorescence changes following displacement of mantATP from pcMyosin by an excess of ATP over 600 s (grey line) with the best fit single exponential superimposed (dashed line). The residual plot is shown below the main plot on an expanded y-scale.

Figure S2. Individual mant-ATP or mant-dATP displacement values for pcHMM, rsHMM, pcS1, and pcMyosin. Each point represents one run for each condition with summary displayed as mean ± s.e.m. Vertical dashed lines separate HMM, S1 and myosin sections. Statistical comparisons were done with one-way ANOVA with Dunnett’s multiple comparisons.

Figure S3. Individual data points from multiple turnover assay with pcHMM. Values for (A) tau, (B) *k*_cat_ and (C) *k*_obs_ for control, mant-dATP, and 0.5μM Mava with summary displayed as mean ± s.e.m. Statistical comparisons were done with one-way ANOVA with Dunnett’s multiple comparisons.

Figure S4. SDS-PAGE of pcHMM before and after RLC exchange and rskHMM and pcS1. This is the same gel from Figure 1E expanded to show rskHMM and pcS1.
